# *Heuchera* Creme Brulee and Mahogany Medicinal Value under Water Stress and Oligosaccharide (COS) Treatment

**DOI:** 10.1155/2019/4242359

**Published:** 2019-02-17

**Authors:** Hosam O. Elansary, Amal M. E. Abdel-Hamid, Eman A. Mahmoud, Fahed A. Al-Mana, Diaa O. El-Ansary, Tarek K. Zin El-Abedin

**Affiliations:** ^1^Plant Production Department, College of Food and Agriculture Sciences, King Saud University, P.O. Box 2460, Riyadh 11451, Saudi Arabia; ^2^Floriculture, Ornamental Horticulture and Garden Design Department, Faculty of Agriculture (El-Shatby), Alexandria University, Alexandria, Egypt; ^3^Department of Geography, Environmental Management and Energy Studies, University of Johannesburg, APK Campus, 2006, South Africa; ^4^Department of Biological and Geological Sciences, Faculty of Education, Ain Shams University, Cairo, Egypt; ^5^Department of Food Industries, Damietta University, Damietta, Egypt; ^6^Precision Agriculture Laboratory, Department of Pomology, Faculty of Agriculture (El-Shatby), Alexandria University, Alexandria, Egypt; ^7^Department of Agricultural Engineering, College of Food and Agriculture Sciences, King Saud University, Riyadh, Saudi Arabia

## Abstract

Food borne pathogens cause serious human illnesses and diseases and their control using natural bioactive compounds becomes essential for the progress of agricultural and food industries. Developing novel tools to enhance the medicinal values of traditional horticultural medicinal crops is one of the promising methods for achieving food borne pathogens control. In this study, oligosaccharide water solutions were applied to* Heuchera *Creme Brulee and Mahogany subjected to a normal irrigation interval (2 days) or to prolonged irrigation intervals (6 days) for 6 weeks. Plant morphological, physiological, and metabolic markers associated with the bioactivity of leaf extracts against selected microbes. Oligosaccharide-treated plants showed significant increases in all morphological parameters during normal and prolonged irrigation intervals as compared to those of the controls. Morphological improvement associated with a significant increase in chlorophyll, carbohydrates, proline, K, Ca, phenols, and free and total ascorbate and antioxidants. Superoxide dismutase, catalase, and ascorbate peroxidase activities were higher, while H_2_O_2_ accumulated to a lower extent in oligosaccharide-treated plants. These morphological and metabolic changes associated with increased antibacterial and antifungal activities of leaf extracts and their activities were comparable to antibiotics and antifungal agents (minimum inhibitory concentrations values were 0.5 -0.20 mg^−1^mL for bacteria and 0.08 -0.20 mg^−1^mL for fungi in Mahogany). The application of oligosaccharide and/or water stress might be of great value for producing natural bioactive compounds for food borne pathogens control.

## 1. Introduction

Food borne illnesses such as diarrheal and emetic symptoms are of great importance in the agricultural industry including milk processing worldwide [1, 2]. These illnesses are caused by several microbes such as the bacterial including* Bacillus cereus* [3] and* Listeria monocytogenes* [4, 5] and the severity of the relevant diseases may cause human death. Fungi cause massive agricultural losses and threatens human food storage facilities and produce mycotoxins that cause cancer diseases and neurological disorders. These fungi include* Aspergillus niger* that causes black mold on several horticultural crops and* Aspergillus ochraceus* that contaminate human foods [6–8] and both species developed resistance to antifungal agents [9]. In the same trend, food borne bacteria developed significant resistance to antibiotics [10] which steamed the search for natural alternatives that have more ability to control food borne pathogens. To reduce the losses in the food industry and to maintain the food security, the use of synthetic food preservatives was introduced to the food industry although these preservatives had severe side effects on the human health on the long run [11]. These conditions oriented the search for natural bioactive compounds that have the capabilities to control food borne pathogens.

Horticultural crops tend to produce secondary metabolites during stress conditions such as water stress. Water stress is one of the major limiting factors for agricultural industry, especially in view of the rapidly increasing world population, global climate change, and the increasing worldwide industrial demand for water [12]. Water stress may have several morphological (e.g., leaf number and leaf area), physiological (e.g., carbohydrate and ion composition), metabolic (e.g., SOD activity and composition), and molecular (e.g., free radical scavenging gene products) effects on plants leading to reduced yields as well as increased accumulation of several compounds. Plant metabolic responses to water stress may include the accumulation of carbohydrates [13], increased synthesis of specific proteins, increased stress related nutrient uptake (e.g., K), and accumulation of specific antioxidants such as the phenolic compounds and others that neutralize reactive oxygen species (ROS) [14–17].

Efforts to develop novel tools to enable horticultural crops to cope with water stress on plants are a growing concern worldwide, such as the use of biochar [18], *β*-aminobutyric acid [19], trinexapac-ethyl [20], seaweed extracts [21], nanoparticles [22], and oligosaccharide. Oligosaccharide is a biostimulant produced commercially by subjecting chitin to high temperature, followed by deacetylation using alkaline conditions to remove proteins and calcium [23, 24]. Oligosaccharides may be formulated as a solution or as water-soluble powder. They are widely used as plant elicitors of the production of secondary metabolites [25], particularly polyphenols [26]. Oligosaccharides have also strong antimicrobial activities and may stimulate the growth of beneficiary microbes [27]. Additionally, several studies suggest that it may improve crop yield [23] and enhance stress tolerance [28]. However, little is known regarding the mechanism whereby oligosaccharides enhance water stress tolerance and effect secondary metabolites in horticultural crops.

Saxifragaceae includes 30 genera of herbaceous perennials, such as* Heuchera*, which are known to be genetically diverse due to hybridization [29].* Heuchera* contains about 50 species. One of these is* Heuchera*, which accommodates perennial herbaceous ornamental shade plants widely used in North America, Europe, North Africa, and South Asia [30]. Dozens of colored hybrid cultivars varying in leaf and flower color have been recently introduced in the market. Interestingly, although native people of Europe have used* Heuchera* and other genera of Saxifragaceae as traditional medicinal plants [31] for centuries, the medicinal properties responses of this species to oligosaccharide elicitors under water stress have not been investigated.

In the present study, our objective was to explore the possible effects of oligosaccharides on* Heuchera* grown under normal and prolonged irrigation intervals by using morphological, physiological, and metabolic markers. We hypothesized that stress conditions and oligosaccharides treatment may enhance antimicrobial properties of* Heuchera* plants. The information obtained from this study will contribute to our understanding of oligosaccharides and/or water stress action in plant metabolic responses that may help in the discovery and use of natural bioactive compounds control food spoilage microorganisms.

## 2. Material and Methods

### 2.1. Plant Material and Treatments

Young plants, 10 cm high, of* Heuchera *cultivars Creme Brulee and Mahogany were obtained from local commercial nurseries on January 7^th^, 2017 and 2018. Plants were grown in a polyethylene-covered greenhouse located on the Alexandria-Cairo desert road, Egypt. All plants were identified by Hosam Elansary and registered at the Faculty of Agriculture, Alexandria University, prior to transplanting onto 2.1 L pots containing a mixture of brown peat and perlite (3:1 w/w) supplemented with Crystalon® (20% N: 20% P: 20% K, 2 g/L media). Plants were grown for three weeks under temperatures ranging between 15.1°C and 27.5°C; relative humidity between 58% and 67%; photosynthetically active radiation around 1000 m^−2^ at 12.00 pm; and daily watering of 38-50 mL/plant. Plants were divided into two groups, one of which was watered at 2-day intervals (2DWI), while the other was watered at 6-day intervals (6DWI) for 6 weeks. Oligosaccharide (deacetylation > 95%, MW:‎501.486 g/mol, powder, Aldebeiky Group Co., Cairo, Egypt) water solution was sprayed at concentrations of 50, 200, or 500 ppm until drop off, 2 weeks prior to extending the watering interval; untreated plants were considered as the control treatment. The experiment was laid out in a split-plot design. Irrigation intervals were considered as the main plot and oligosaccharides treatments the subplot. Plants were grouped into three blocks/repetitions (n=3) containing 5 replicates per treatment for a total of 40 plants per cultivar per season in Randomized Complete Block Design (RCBD).

### 2.2. Morphological and Physiological Parameters

Plants were harvested after 6 weeks of stress treatment. At that point, plant height and leaf number were registered. Leaf area was calculated immediately, using a scanner and the AutoCAD program. Total dry weight was determined by drying cleaned plants to constant weight in an oven at 70°C. Total carbohydrates, K^+^, Ca^2+^, and proline were determined in plant leaves at the end of the experiment. Following freeze-drying of samples, they were ground and sieved and then kept at -20°C until further analysis. Total carbohydrates were quantified after Dubios et al. [32] and expressed on a percent basis. One gram of frozen leaves was used to obtain the cell sap, then a dilution (1:100, v/v) was used for the determination of K^+^ and Ca^2+^ concentrations using an inductively coupled plasma spectrophotometer [33]. Proline leaf content was determined in the Department of Plant Production, King Saud University using a spectrophotometer at 520 nm [34, 35].

### 2.3. Antioxidants, Chlorophyll, Phenols, and Enzyme Activities

Air dried leaves were ground into fine powder; 0.25 g of this from each sample was dissolved into 3 mL methanol (99%) while stirring on a magnetic agitator at low speed, in the dark, for 24 h at room temperature. Methanolic extracts were centrifuged for 5 min, under cooling, at 10,000 RPM (7,000 ×* g*); the supernatant (~2.7 mL) was dried in a rotary evaporator to produce a semisolid extract which was stored for later antioxidant analysis. Antioxidant activities of all samples were determined in the Department of Plant Produciton, King Saud University using the 2,2′-diphenylpicrylhydrazyl (DPPH) and *β*-carotene-linoleic acid methods which measure OH^−^ scavenging activities according to Elansary et al. [21]. For the DPPH method, samples were incubated for 30 min, after which absorbance was measured at 517 nm. For the *β*-carotene-linoleic acid assay, absorbance was measured at 470 nm. The sample concentration required to scavenge 50% of DPPH/ *β*-carotene-linoleic acid (IC_50_ in *μ*g/mL) was determined by plotting the inhibition percentage against extract concentration. Butylated hydroxytoluene (BHT) was used as a positive control and experiments were repeated twice in triplicate. Total phenolic content in methanolic leaf extracts were performed using the Folin-Ciocalteau colorimetric method using gallic acid as the reference and expressing the results as gallic acid equivalents (mg GAE g^−1^ ext.) [36, 37]. Total chlorophyll content was quantified in fresh leaves according to Moran and Porath [38].

Ground-frozen leaves were used to quantify total and free ascorbate after Elansary et al. [21]. Briefly, 0.5 g of ground-frozen leaf tissues were homogenized in 8 mL cooled trichloroacetic acid (TCA, 5%, w/v); next, the mixture was centrifuged for 10 min (10,000 ×* g*) at 4°C. The supernatant was incubated with a mixture of PBS (200 mM, pH 7.4) and dithiothreitol (DTT, 1.5 mM) for 50 min; excess DTT was removed by adding N-ethylmaleimide (NEM, 200 *μ*L, 0.5%, w/v). The solution was then mixed with TCA (1 mL, 10%, w/v), o-phosphoric acid (800 *μ*L, 42%, w/v), and 2,2-dipyridyl in 70% (v/v) ethanol (800 *μ*L 65 mM) and iron(III) chloride (400 *μ*L, 3%, w/v) and incubated for 1 h at 42°C. Absorbance by the mixture was measured at 525 nm. Free ascorbate was determined using the same procedure, except DTT and NEM were replaced with 400 *μ*L deionized water, while free and total ascorbate contents were determined using standard curves.

Catalase (CAT), ascorbate peroxidase (APX), and superoxide dismutase (SOD) activities as well as H_2_O_2_ accumulation were quantified in leaves tissues following Elansary et al. [21].

### 2.4. Microorganisms and Medicinal Properties

The medicinal properties of methanolic leaf extracts were studied against selected pathogenic bacteria and fungi. The selected bacteria were* Listeria monocytogenes* (clinical isolate),* Bacillus cereus *(ATCC 14579),* Staphylococcus aureus *(ATCC 6538),* Micrococcus flavus* (ATCC 10240),* Pseudomonas aeruginosa* (ATCC 27853), and* Escherichia coli* (ATCC 35210). The selected fungi were* Aspergillus niger* (ATCC 6275),* A. ochraceus* (ATCC 12066),* A. flavus* (ATCC 9643),* Penicillium ochrochloron* (ATCC 48663), and* Candida albicans* (ATCC 12066). The microdilution method [39] was used to determine the antibacterial and antifungal activities. In the antibacterial assay, the minimum inhibitory bactericidal concentration (MIC) was defined as the lowest concentration resulting in growth stop of the bacteria at the binocular level. The minimum bactericidal concentration (MBC) was defined as the lowest concentration resulting in killing 99.5% of the original inoculum. Also, the MBC was determined by serial subcultivation of the bacterial using 0.1-0.2 mg/mL of bacterial solution added to 100 *μ*L of TSB and incubated for one day. In the antifungal activity assay, the minimum inhibitory concentration (MIC) was defined as the lowest concentration inhibiting the fungal growth at the binocular level while the minimum fungicidal concentration (MFC) was determined using subcultivations of the fungi (0.1-4.0 mg/mL) and was defined as the concentration killing 99.5% of the original inoculum. Experiments were performed twice and negative controls (5% DMSO) as well as positive controls [antibacterial assay, streptomycin and ampicillin, 0.01-10 mg/mL; antifungal, Fluconazole (FLZ) and ketoconazole (KLZ)] were used. Experiments were repeated twice.

### 2.5. Statistical Analyses

The data obtained during the two growing seasons in 2017 and 2018 were expressed as means and Least Significant Difference (LSD) was determined using the one way ANOVA test in SPSS (PASW Ver. 21) at* P *≤ 0.05.

## 3. Results

### 3.1. Morphological and Physiological Responses to Irrigation Intervals and Oligosaccharide

Increasing watering intervals from 2 to 6 days significantly reduced morphological parameters in both* Heuchera* cultivars tested, including leaf number, leaf area, plant dry weight, and plant height (Table 1). Interestingly, under the normal irrigation interval (2DWI), the application of the oligosaccharide at 50 and 200 ppm significantly increased leaf number and area, plant dry weight, and plant height in both cultivars treated plants in both seasons, compared to untreated plants. Further, under prolonged irrigation interval (6DWI), there were significant increases in both Creme Brulee and Mahogany in all morphological parameters measure, in plants treated with oligosaccharide at 50 and 200 ppm, compared to oligosaccharide at 500 ppm and control treatment. Prolonged irrigation interval (6DWI) significantly reduced total carbohydrates, K, Ca, and proline contents in plants of both, Creme Brulee and Mahogany, compared to the normal irrigation interval (2DWI) as shown in Table 2. Under 2DWI as well as 6DWI, total carbohydrates, K, Ca, and proline contents increased significantly in the leaves of oligosaccharides -treated plants at 50 and 200 ppm, compared to controls and 500 ppm oligosaccharide treatment, in both growing seasons.

### 3.2. General Antioxidants, Phenolics, and Chlorophylls

Extension of irrigation interval from 2 to 6 days caused a significant increase in DPPH free radical scavenging activity in both* Heuchera* cultivars (Table 3). The DPPH (IC_50_) of Creme Brulee plants decreased in the first season (2017), which indicates an increase in scavenging activity; a similar pattern was observed in the second season. Furthermore, there was a significant increase in scavenging activity of leaf extracts following water stress conditions, as revealed by the *β*-Carotene-linoleic acid assay.* Heuchera* plants (Creme Brulee and Mahogany) growing under normal irrigation conditions (2DWI) as well as prolonged irrigation (6DWI) showed a significant increase in scavenging activity by leaf extracts following application of oligosaccharides at 50 and 200 ppm, compared to controls and 500 ppm oligosaccharide treatment in both the 2017 and 2018 years. Creme Brulee plants treated with 200 ppm oligosaccharide showed increased DPPH (IC_50_) free radical scavenging activity in plants subjected to 2 and 6 days irrigation intervals in the 2017 season.

Similarly, there was a significant increase in total phenolic content in plants of both cultivars tested, upon widening of the irrigation interval, in the two growing seasons under study (Table 3). Interestingly, oligosaccharide treatments boosted phenolic content, particularly in plants of both cultivars treated with 50 and 200 ppm. In 2017, Creme Brulee leaf extracts showed an increase in phenolic content in plants subjected to 2DWI and 6DWI, respectively. Similarly, the same year Mahogany leaf extracts showed an increase in phenolic content in plants subjected to 2DWI and 6DWI, respectively. Total phenolic content increased significantly in plants treated with 50 and 200 ppm oligosaccharide, compared to the control and 500 ppm oligosaccharide treatments. Total chlorophyll content in Creme Brulee and Mahogany was significantly reduced in control plants subjected to 6DWI. In contrast, application of oligosaccharide showed significant increase in chlorophyll content of treated plants at 50 and 200 ppm, compared to control and 500 ppm oligosaccharide, under both watering intervals, in both cultivars and in the two growth seasons evaluated. In summary, antioxidant activity and phenolic and chlorophyll contents were higher in Mahogany than in Creme Brulee, in the two seasons under study.

### 3.3. Enzymatic and Nonenzymatic Antioxidants

**Figure 1:**
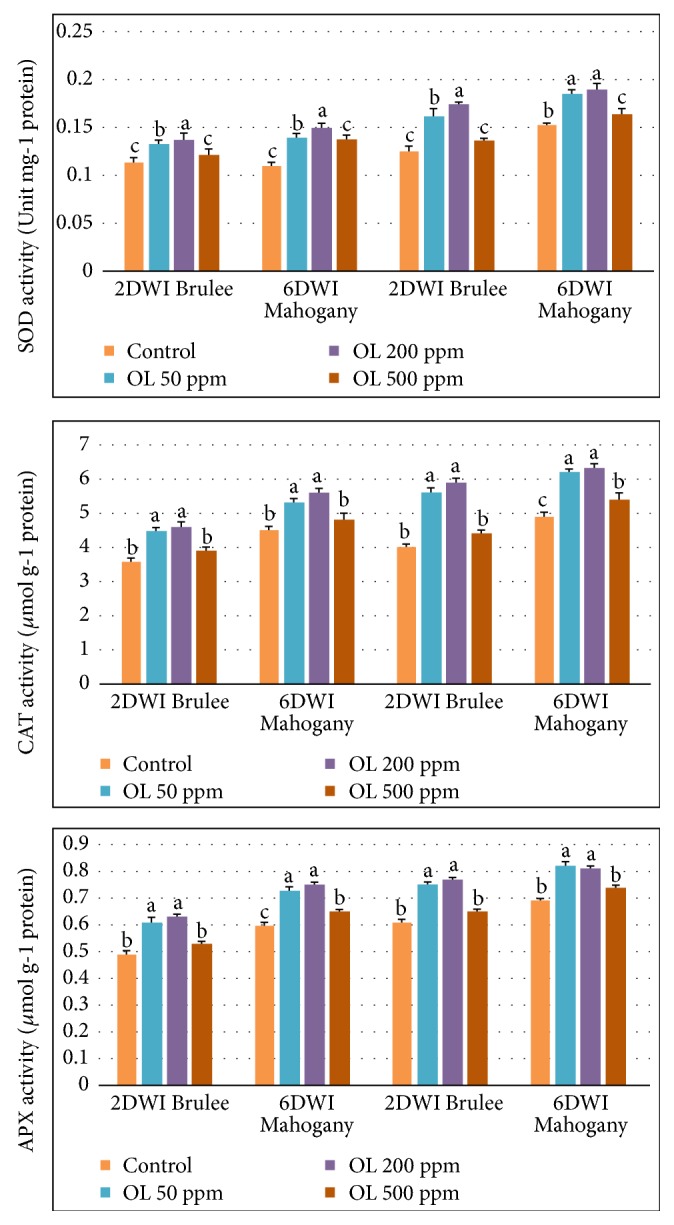
SOD, CAT, and APX activities in* Heuchera* subjected to prolonged irrigation intervals and different oligosaccharides (OL) concentrations.

Major antioxidant SOD, CAT, and APX enzyme activities showed significant increases in Creme Brulee and Mahogany plants subjected to oligosaccharide treatments at 50 and 200 ppm, compared to oligosaccharides at 500 ppm and control treatments under normal and prolonged irrigation intervals (Figure 1). In both cultivars, application of oligosaccharide at 200 ppm resulted in the highest SOD, CAT, and APX enzyme activities recorded both under 2DWI and 6DWI and in both seasons studied. Mahogany plants showed slightly higher values of SOD, CAT, and APX enzymes activities compared to Creme Brulee.

**Figure 2:**
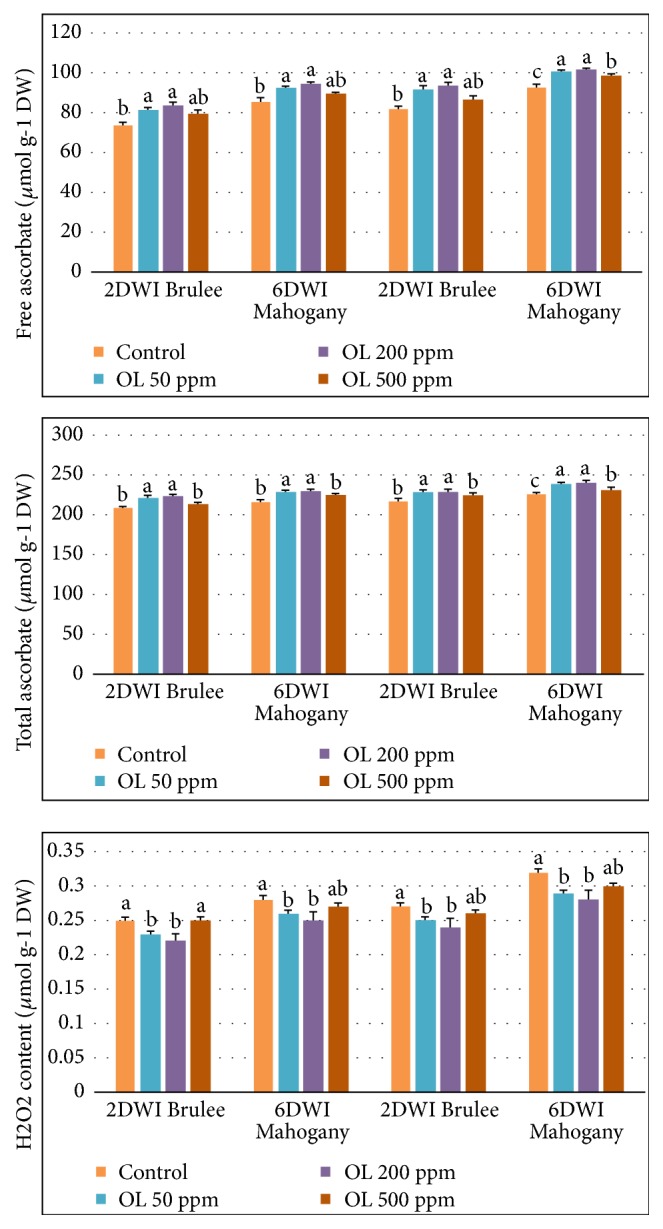
Free and total ascorbate and H_2_O_2_ content in* Heuchera* plants subjected to prolonged irrigation intervals and different oligosaccharides (OL) concentrations.

Free and total ascorbate (nonenzymatic antioxidants) showed a significant increase in oligosaccharides-treated plants at 50 and 200 ppm compared to oligosaccharide at 500 ppm and control treatments under normal and prolonged irrigation intervals (Figure 2). Concomitantly, there were significant reductions in H_2_O_2_ content in oligosaccharides-treated plants at 50 and 200 ppm, compared to the 500 ppm dose as well as the control treatment, in both cultivars and in both seasons (Figure 2).

### 3.4. Antibacterial and Antifungal Activities

*Heuchera *Creme Brulee leaf extracts showed antibacterial activities against screened bacteria as shown in Table 4. The highest antibacterial activities were found in plants subjected to prolonged irrigation intervals and 200/500 ppm oligosaccharide. In Mahogany plants, there were higher antibacterial activities of leaf extracts against the same collection of bacteria. The highest antibacterial activities were against* B. cereus* and* M. flavus* in plants treated with prolonged irrigation intervals and 500 ppm oligosaccharide. Both cultivars leaf extracts showed comparable antibacterial activities to antibiotics under stress and oligosaccharides treatments.

The antifungal activities of* Heuchera* cultivars leaf extracts were investigated as shown in Table 5. Creme Brulee showed antifungal activities as well as Mahogany. In both cultivars, prolonged irrigation and oligosaccharide treatments (500 and 200ppm) showed the highest antifungal activities. The antifungal activities of Mahogany leaf extracts were higher than Creme Brulee and were comparable to antibiotics.

## 4. Discussion

A significant reduction in morphological parameters, such as plant height, number of leaves, leaf area, and plant dry weight, due to extension of the irrigation interval which is in agreement with previous studies [20, 40–42]. These morphological changes associated with major physiological alterations, such as changes in carbohydrate, K, Ca, proline, chlorophylls, and antioxidants contents [15, 21, 42]. Oligosaccharide sprays at specific doses enhanced the growth of the two* Heuchera* cultivars tested here during normal and extended irrigation intervals, as reflected by increased vegetative growth. Similar observations have been described before for oligosaccharide treatments on dry matter and essential oil yield in* Thymus daenensis* Celak [28]. In that study, the authors suggested that the increase in dry matter and in the essential oil yield under mild stress might be attributed to increased proline content and to lipid peroxidation.

Accumulation of carbohydrates might be an important indicator of stress tolerance in plants by means of osmotic adjustment and scavenging of ROS [43, 44]. Additionally, the accumulation of proline balances vacuolar ion osmotic pressure [20, 40] and maintains water influx [45]. Proline accumulation increased under an extended irrigation interval in the present study, an original contribution of the study reported herein is that we report the increase in leaf proline content at normal irrigation interval, something not previously reported, using low doses of 50 and 200 ppm oligosaccharide. The accumulation of K and Ca ions in plant leaves is a well-known mechanism of osmotic adjustment during stress conditions, such as drought and salinity. This accumulation of K and Ca is associated with carbohydrate accumulation in stressed plants, which enhances plant performance during stress and improves cell turgor pressure [21, 40]. Interestingly, K and Ca accumulation in plant during stress conditions enhance photosynthetic rate, leading to increased chlorophyll content (drought resistance mechanism) as well as carbohydrate accumulation, such as documented herein, which helped in improving plant performance during stress. The application of oligosaccharide at low rate significantly increased leaf K and Ca content and helped in attaining osmotic adjustment during water stress. Such accumulation of K and Ca in plants might be associated with antifungal activities [46–48].

Excess ROS, e.g., H_2_O_2_, O_2_, and OH^−^, are produced in plants under water stress conditions, due to imbalance between production and utilization of electrons. This condition may cause damage and even cell death [49], if ROS are not effectively removed. An antioxidant defense mechanism in plants consists of enzymatic and nonenzymatic tools that intervene to maintain the intracellular redox balance under conditions of stress. Nonenzymatic tools include secondary metabolites, such as total and free ascorbate, as well as phenols and their derivatives (e.g., flavanones and anthocyanins) [21, 50, 51]. Enzymatic tools include many enzymes, among which, the most common are SOD, CAT, and APX, which control H_2_O_2_ production in plants [44, 50]. Further, these compounds including ascorbate (derivative of ascorbic acid) have well-known antibacterial and antifungal activities as found in this study [52–55]. In the current study we found strong antibacterial and antifungal activities in plants with accumulated ascorbate as in plants subjected to prolonged and oligosaccharide treatments.

We observed a significant increase in leaves phenolic composition following water stress conditions, which became higher in oligosaccharides-treated plants. This increase in total phenolic content in leaves was reflected in an increase in antioxidant activity, as determined by the DPPH and linoleic acid assays. Additionally, oligosaccharide doses of 50 and 200 ppm caused a significant increase in phenolic content in leaves, compared to control plants; these results are consistent with those reported by [26], who reported higher polyphenols in plants of* Origanum vulgare* subjected to oligosaccharide treatment. Phenols play an important role in removing ROS in stressed plants and largely affect the antioxidant estimations of DPPH and linoleic acid assays, which mainly measure OH^−^ free radical. The higher antibacterial and antifungal activities found in both cultivars leaf extracts in plants subjected to prolonged irrigation intervals and oligosaccharide treatments are associated with the accumulation of phenols in treated plants. The accumulation of phenols has important inhibiting effects on the growing of bacteria and fungi [56, 57]. It was also clear that Creme Brulee and Mahogany differed from one another with respect to morphological (e.g., plant height), physiological parameters (e.g., phenolic composition and antioxidant), and biochemical activities (antibacterial and antifungal activities). Such variation between cultivars has been documented for other species in response to stress and genetics [58, 59].

## 5. Conclusion

This is the first report on enhancing* Heuchera* plants medicinal values by subjecting the plants to water stress and oligosaccharide treatments. Morphological, physiological, and antimicrobial parameters were studied to document the interaction between stress tolerance and oligosaccharide applications in* Heuchera* plants subjected to water stress and oligosaccharide treatments. The study revealed that oligosaccharide foliar application effectively ameliorated water stress deleterious effects on plant growth, enhanced the phytochemical composition, and improved the medicinal values of treated plants. These findings suggest that water stress accompanied by oligosaccharide sprays might be a valuable tool in improving the medicinal value in horticultural crops and the future development of novel tools to control food borne pathogens and respective microbial diseases.

## Figures and Tables

**Table 1:** Effect of water deficit and oligosaccharides treatment on leaf number, leaf area, plant dry weight, and plant height in two *Heuchera *cultivars after six weeks of treatment initiation. Values are expressed as means (± sd).

Water interval	Oligosaccharides treatment (ppm)		Leaf number (leaf plant^−1^)		Leaf area (cm^2^ plant^−1^)		Plant dry weight (g plant^−1^)		Plant height (cm)	
			2017	2018	2017	2018	2017	2018	2017	2018
2DWI	0	Creme Brulee	15.6 ± 0.2b^*∗*^	15.2 ± 0.1b	651.2 ± 15.1b	648.2 ± 11.1b	11.2 ± 0.1b	11.2 ± 0.2b	29.1 ± 0.1b	28.8 ± 0.2b
	50		16.1 ± 0.1ab	16.1 ± 0.2a	690.1 ± 13.1a	686.3 ± 14.5a	12.3 ± 0.1a	12.1 ± 0.2a	33.2 ± 0.1a	30.8 ± 0.3a
	200		17.1 ± 0.4a	17.2 ± 0.1a	703.1 ± 14.3a	699.2 ± 15.1a	12.4 ± 0.1a	12.2 ± 0.2a	32.4 ± 0.2a	30.7 ± 0.2a
	500		15.6 ± 0.1b	15.3 ± 0.2b	639.2 ± 11.1b	650.5 ± 22.3b	11.3 ± 0.1b	11.2 ± 0.2b	30.1 ± 0.1b	29.4 ± 0.1b
6DWI	0		7.0 ± 0.1d	7.1 ± 0.2d	303.1 ± 10.3d	311.1 ± 13.1d	5.5 ± 0.1d	5.6 ± 0.1d	17.3 ± 0.3d	16.8 ± 0.1d
	50		8.6 ± 0.0cd	8.5 ± 0.3cd	350.9 ± 15.1c	358.1 ± 11.2c	6.3 ± 0.1c	6.2 ± 0.1c	19.4 ± 0.1c	18.8 ± 0.1c
	200		9.0 ± 0.1c	9.1 ± 0.2c	361.3 ± 14.1c	351.6 ± 17.5c	6.1 ± 0.2c	6.2 ± 0.1c	19.5 ± 0.1c	19.0 ± 0.1c
	500		7.3 ± 0.0d	7.2 ± 0.1d	311.2 ± 13.1d	306.1 ± 14.1d	5.3 ± 0.1d	5.3 ± 0.1d	17.4 ± 0.2cd	17.2 ± 0.1d

2DWI	0	Mahogany	13.2 ± 0.1b	12.8 ± 0.1b	516.1 ± 22.1b	512.5 ± 10.1b	10.8 ± 0.3b	10.7 ± 0.1b	30.8 ± 0.3b	31.1 ± 0.4b
	50		14.4 ± 0.1a	14.2 ± 0.2a	563.1 ± 20.1a	567.5 ± 11.2a	11.6 ± 0.1a	11.7 ± 0.2a	33.3 ± 0.1a	32.9 ± 0.3a
	200		14.7 ± 0.3a	14.2 ± 0.3a	573.1 ± 10.3a	578.1 ± 12.1a	11.6 ± 0.1a	11.7 ± 0.1a	34.2 ± 0.4a	33.2 ± 0.3a
	500		13.2 ± 0.1b	13.1 ± 0.1b	510.3 ± 11.3b	502.1 ± 16.7b	10.8 ± 0.1b	10.8 ± 0.2b	31.4 ± 0.1b	30.7 ± 0.1b
6DWI	0		6.1 ± 0.2e	6.2 ± 0.2e	220.1 ± 12.1d	215.3 ± 15.2d	5.4 ± 0.1d	5.5 ± 0.1d	18.1 ± 0.1d	18.3 ± 0.3d
	50		7.4 ± 0.1d	7.1 ± 0.1d	261.1 ± 13.1c	268.3 ± 11.3c	6.3 ± 0.1c	6.2 ± 0.1c	21.2 ± 0.3c	20.7 ± 0.1c
	200		8.1 ± 0.1c	8.2 ± 0.2c	271.3 ± 10.1c	277.3 ± 11.1c	6.3 ± 0.1c	6.3 ± 0.1c	20.5 ± 0.2c	20.9 ± 0.1c
	500		6.2 ± 0.1e	6.1 ± 0.1e	210.2 ± 11.5d	221.5 ± 15.1d	5.5 ± 0.1d	5.4 ± 0.1d	18.3 ± 0.1d	18.7 ± 0.3d

^*∗*^ Means followed by different letters within columns are significantly different, based on LSD test (*P ≤ 0.05*).

**Table 2:** Effect of irrigation intervals and oligosaccharides treatment on total carbohydrate, K, Ca, and proline content in the leaves oftwo *Heuchera *cultivars in two successive seasons. Values are means (± sd).

Water interval	Oligosaccharides treatment (ppm)		Total carbohydrates (% DW)		K (mg g^−1^ DW)		Ca (mg g^−1^ DW)		Proline (mg g^−1^ DW)	
			2017	2018	2017	2018	2017	2018	2017	2018
2DWI	0	Creme Brulee	13.45 ± 0.1b^*∗*^	13.37 ± 0.1b	19.7 ± 0.1d	19.5 ± 0.5d	3.76 ± 0.05b	3.63 ± 0.04b	1.35 ± 0.05c	1.32 ± 0.01c
	50		14.33 ± 0.1a	14.22 ± 0.1ab	24.8 ± 0.1b	23.9 ± 0.1b	4.12 ± 0.0a	4.11 ± 0.2a	1.44 ± 0.03b	1.41 ± 0.00cb
	200		14.53 ± 0.2a	14.67 ± 0.1a	25.8 ± 0.2b	24.9 ± 0.1b	4.15 ± 0.09a	4.09 ± 0.03a	1.47 ± 0.01b	1.44 ± 0.03b
	500		13.53 ± 0.1b	13.49 ± 0.1b	19.9 ± 0.0d	19.4 ± 0.1d	3.85 ± 0.01b	3.79 ± 0.05b	1.37 ± 0.01c	1.35 ± 0.02c
6DWI	0		12.19 ± 0.2c	12.05 ± 0.1c	21.5 ± 0.1b	21.3 ± 0.3c	3.63 ± 0.06b	3.65 ± 0.06b	1.48 ± 0.02b	1.46 ± 0.01ab
	50		12.89 ± 0.1cb	12.51 ± 0.1c	27.3 ± 0.3a	26.8 ± 0.1a	4.05 ± 0.03a	3.97 ± 0.01a	1.56 ± 0.03a	1.53 ± 0.03a
	200		12.87 ± 0.1cb	12.98 ± 0.1bc	27.9 ± 0.1a	27.7 ± 0.1a	4.19 ± 0.07a	4.11 ± 0.04a	1.58 ± 0.02a	1.55 ± 0.02a
	500		12.32 ± 0.2c	12.31 ± 0.1c	22.2 ± 0.2c	21.8 ± 0.2c	3.78 ± 0.06b	3.70 ± 0.05b	1.49 ± 0.03ab	1.47 ± 0.01ab

2DWI	0	Mahogany	15.32 ± 0.1b^a^	15.05 ± 0.1b	21.3 ± 0.1c	21.7 ± 0.3c	3.61 ± 0.04b	3.67 ± 0.08b	1.42 ± 0.02c	1.38 ± 0.01c
	50		15.99 ± 0.1ab	15.91 ± 0.0a	26.4 ± 0.1b	26.1 ± 0.1b	3.97 ± 0.03a	3.94 ± 0.05a	1.55 ± 0.04b	1.46 ± 0.01b
	200		16.30 ± 0.1a	16.13 ± 0.3a	26.8 ± 0.1b	26.6 ± 0.3b	4.06 ± 0.06a	4.01 ± 0.06a	1.58 ± 0.08b	1.49 ± 0.04b
	500		15.51 ± 0.1b	15.27 ± 0.3b	23.3 ± 0.3d	22.6 ± 0.1c	3.73 ± 0.04b	3.75 ± 0.03b	1.47 ± 0.01c	1.41 ± 0.01c
6DWI	0		14.41 ± 0.2c	14.12 ± 0.3c	25.5 ± 0.1b	24.7 ± 0.1b	3.58 ± 0.02b	3.68 ± 0.02b	1.56 ± 0.03b	1.51 ± 0.04b
	50		15.45 ± 0.1b	15.13 ± 0.1b	29.9 ± 0.1a	28.9 ± 0.3a	3.93 ± 0.01a	3.95 ± 0.01a	1.70 ± 0.01a	1.69 ± 0.02a
	200		15.55 ± 0.1b	15.25 ± 0.5b	30.3 ± 0.1a	29.7 ± 0.2a	3.93 ± 0.01a	3.96 ± 0.04a	1.74 ± 0.03a	1.71 ± 0.02a
	500		14.37 ± 0.1c	14.24 ± 0.3c	26.4 ± 0.1a	25.8 ± 0.3b	3.63 ± 0.05b	3.67 ± 0.02b	1.59 ± 0.02b	1.54 ± 0.01b

^*∗*^ Means followed by different letters within columns are significantly different, based on LSD test (*P ≤ 0.05)*.

**Table 3:** Antioxidant activity in leaf methanolic extracts, total phenolic and total chlorophyll content of two *Heuchera *cultivars. Values are means of triplicate determinations ±sd.

Water interval	Oligosaccharides treatment (ppm)		DPPH free radical scavenging activity (IC50, *μ*g ml^−1^)		*β*-Carotene-linoleic acid assay (IC_50_, *μ*g ml^−1^)		Total phenolic content (mg GAE g^−1^)		Total chlorophyll content (mg g^−1^ DW)	
			2017	2018	2017	2018	2017	2018	2017	2018
2DWI	0	Creme Brulee	10.3 ± 0.01a^*∗*^	11.1 ± 0.07a	11.2 ± 0.01a	11.5 ± 0.01a	10.4 ± 0.1 c	9.7 ± 0.1 c	0.65 ± 0.04b	0.63 ± 0.01bc
	50		9.3 ± 0.06b	9.9 ± 0.01b	10.3 ± 0.03b	10.4 ± 0.02b	10.9 ± 0.1 b	10.4 ± 0.3 b	0.69 ± 0.02a	0.67 ± 0.03a
	200		9.1 ± 0.05b	9.9 ± 0.02b	10.3 ± 0.03b	10.7 ± 0.03b	10.8 ± 0.0 b	10.5 ± 0.2 b	0.70 ± 0.02a	0.68 ± 0.01a
	500		9.6 ± 0.03a	10.4 ± 0.03a	11.2 ± 0.02a	11.5 ± 0.04a	10.4 ± 0.2 c	10.0 ± 0.1 c	0.68 ± 0.01ab	0.65 ± 0.02ab
6DWI	0		8.2 ± 0.04c	8.9 ± 0.05c	9.3 ± 0.01c	10.1 ± 0.03c	10.9 ± 0.4 b	10.4 ± 0.2 b	0.61 ± 0.02c	0.60 ± 0.03c
	50		6.3 ± 0.02d	6.8 ± 0.00d	7.4 ± 0.02d	8.2 ± 0.02d	11.6 ± 0.3 a	11.2 ± 0.2 a	0.65 ± 0.01b	0.64 ± 0.02b
	200		6.2 ± 0.01d	6.8 ± 0.01d	7.2 ± 0.03d	8.2 ± 0.02d	11.6 ± 0.1 a	11.3 ± 0.2 a	0.65 ± 0.01b	0.65 ± 0.03ab
	500		7.8 ± 0.03c	8.3 ± 0.01c	9.2 ± 0.04c	9.9 ± 0.01c	11.1 ± 0.2 b	10.5 ± 0.4 b	0.62 ± 0.02c	0.61 ± 0.01c

2DWI	0	Mahogany	8.9 ± 0.4a	9.6 ± 0.1a	10.1 ± 0.3a	10.7 ± 0.2a	12.3 ± 0.2 c	11.7 ± 0.3 c	0.71 ± 0.01b	0.70 ± 0.02b
	50		7.4 ± 0.02b	7.9 ± 0.01b	8.5 ± 0.01b	9.4 ± 0.03b	12.7 ± 0.3 b	12.4 ± 0.1 b	0.75 ± 0.02a	0.74 ± 0.01a
	200		7.1 ± 0.01b	7.8 ± 0.04b	8.4 ± 0.00b	9.3 ± 0.03b	12.9 ± 0.4 b	12.5 ± 0.2 b	0.76 ± 0.01a	0.75 ± 0.01a
	500		8.2 ± 0.02a	8.8 ± 0.03a	9.5 ± 0.03a	10.4 ± 0.02a	12.2 ± 0.1 c	12.1 ± 0.3 bc	0.72 ± 0.01b	0.70 ± 0.02b
6DWI	0		7.1 ± 0.03b	7.7 ± 0.02b	8.4 ± 0.01b	8.9 ± 0.03b	13.1 ± 0.1 b	12.4 ± 0.2 b	0.66 ± 0.02c	0.63 ± 0.01c
	50		5.4 ± 0.01c	5.7 ± 0.02c	6.6 ± 0.03c	7.2 ± 0.03c	13.8 ± 0.2 a	13.2 ± 0.0 a	0.70 ± 0.01c	0.68 ± 0.03c
	200		4.8 ± 0.03c	5.5 ± 0.07c	6.2 ± 0.02c	7.1 ± 0.02c	13.9 ± 0.3 a	13.4 ± 0.1 a	0.71 ± 0.01a	0.68 ± 0.02a
	500		6.8 ± 0.01b	7.4 ± 0.03b	7.9 ± 0.03b	8.2 ± 0.01b	13.4 ± 0.2 ab	12.5 ± 0.1b	0.68 ± 0.02b	0.64 ± 0.03b

^*∗*^ Means followed by different letters within columns are significantly different based on LSD test (*P ≤ 0.05*).

**Table 4:** Minimum inhibitory (MIC) and bactericidal concentration (MBC) of *Heuchera *Creme Brulee and Mahogany leaf extracts (mg^−1^mL) for the 2018 growing season.

Water interval	Oligosaccharides treatment (ppm)		*Escherichia coli*	*Staphylococcus aureus *	*Bacillus cereus*	*Micrococcus flavus*	*Pseudomonas aeruginosa*	*Listeria monocytogenes*
2DWI	0	Creme Brulee	0.23 ± 0.01	0.14 ± 0.01	0.10 ± 0.02	0.11 ± 0.01	0.13 ± 0.02	0.20 ± 0.01
			0.45 ± 0.01	0.33 ± 0.03	0.21 ± 0.01	0.22 ± 0.02	0.27 ± 0.01	0.40 ± 0.01
	200		0.21 ± 0.03	0.13 ± 0.02	0.9 ± 0.04	0.10 ± 0.01	0.12 ± 0.01	0.19 ± 0.02
			0.42 ± 0.01	0.31 ± 0.01	0.18 ± 0.01	0.20 ± 0.01	0.24 ± 0.01	0.37 ± 0.01
	500		0.19 ± 0.05	0.12 ± 0.03	0.8 ± 0.02	0.9 ± 0.03	0.11 ± 0.02	0.18 ± 0.01
			0.40 ± 0.01	0.30 ± 0.01	0.17 ± 0.01	0.19 ± 0.01	0.23 ± 0.01	0.35 ± 0.01
6DWI	0		0.20 ± 0.05	0.12 ± 0.06	0.9 ± 0.01	0.10 ± 0.01	0.11 ± 0.01	0.19 ± 0.02
			0.40 ± 0.01	0.29 ± 0.01	0.18 ± 0.01	0.20 ± 0.01	0.24 ± 0.01	0.37 ± 0.01
	200		0.18 ± 0.01	0.11 ± 0.01	0.7 ± 0.03	0.9 ± 0.02	0.10 ± 0.01	0.17 ± 0.02
			0.39 ± 0.01	0.27 ± 0.01	0.17 ± 0.01	0.19 ± 0.01	0.21 ± 0.01	0.33 ± 0.01
	500		0.17 ± 0.01	0.10 ± 0.04	0.6 ± 0.04	0.8 ± 0.01	0.9 ± 0.02	0.15 ± 0.04
			0.38 ± 0.01	0.23 ± 0.01	0.15 ± 0.01	0.16 ± 0.00	0.18 ± 0.01	0.30 ± 0.01

2DWI	0	Mahogany	0.20 ± 0.4	0.12 ± 0.3	0.9 ± 0.3	0.10 ± 0.01	0.11 ± 0.02	0.17 ± 0.03
			0.40 ± 0.01	0.28 ± 0.01	0.17 ± 0.01	0.20 ± 0.01	0.24 ± 0.01	0.33 ± 0.01
	200		0.18 ± 0.01	0.11 ± 0.04	0.8 ± 0.00	0.9 ± 0.03	0.10 ± 0.02	0.16 ± 0.02
			0.38 ± 0.01	0.25 ± 0.01	0.16 ± 0.01	0.19 ± 0.01	0.21 ± 0.01	0.31 ± 0.01
	500		0.17 ± 0.01	8.10 ± 0.03	0.7 ± 0.03	0.8 ± 0.02	0.09 ± 0.01	0.15 ± 0.03
			0.36 ± 0.01	0.23 ± 0.01	0.14 ± 0.01	0.16 ± 0.01	0.18 ± 0.01	0.30 ± 0.01
6DWI	0		0.18 ± 0.01	0.10 ± 0.02	0.8 ± 0.01	0.8 ± 0.04	0.10 ± 0.01	0.16 ± 0.02
			0.38 ± 0.01	0.23 ± 0.01	0.16 ± 0.01	0.16 ± 0.01	0.20 ± 0.01	0.31 ± 0.01
	200		0.16 ± 0.03	0.9 ± 0.07	0.7 ± 0.02	0.7 ± 0.02	0.9 ± 0.03	0.15 ± 0.01
			0.35 ± 0.01	0.20 ± 0.01	0.14 ± 0.01	0.14 ± 0.02	0.18 ± 0.01	0.30 ± 0.01
	500		0.15 ± 0.01	0.7 ± 0.03	0.5 ± 0.03	0.6 ± 0.01	0.8 ± 0.02	0.13 ± 0.01
			0.33 ± 0.01	0.18 ± 0.01	0.12 ± 0.01	0.12 ± 0.01	0.16 ± 0.01	0.27 ± 0.01

	Streptomycin		0.9 ± 0.01	0.20 ± 0.01	0.05 ± 0.01	0.10 ± 0.005	0.07 ± 0.00	0.16 ± 0.01
			0.42 ± 0.01	0.43 ± 0.01	0.14 ± 0.01	0.19 ± 0.005	0.14 ± 0.01	0.33 ± 0.01
	Ampicillin		0.24 ± 0.01	0.10 ± 0.03	0.10 ± 0.005	0.10 ± 0.002	0.14 ± 0.01	0.16 ± 0.01
			0.44 ± 0.01	0.15 ± 0.01	0.18 ± 0.005	0.16± 0.005	0.22 ± 0.01	0.28 ± 0.01

**Table 5:** Minimum inhibitory (MIC) and fungicidal concentration (MFC) of *Heuchera *Creme Brulee and Mahoganyleaf extracts (mg^−1^mL).

Water interval	Oligosaccharides treatment (ppm)		*Aspergillus niger* MIC MFC	*Aspergillus ochraceus* MIC MFC	*Aspergillus flavus* MIC MFC	*Penicillium ochrochloron * MIC MFC	*Candida albicans* MIC MFC
2DWI	0	Creme Brulee	0.20 ± 0.01	0.21 ± 0.01	0.13 ± 0.02	0.25 ± 0.01	0.14 ± 0.02
			0.42 ± 0.01	0.43 ± 0.03	0.27 ± 0.01	0.53 ± 0.02	0.27 ± 0.01
	200		0.20 ± 0.03	0.19 ± 0.02	0.12 ± 0.01	0.23 ± 0.01	0.12 ± 0.01
			0.41 ± 0.01	0.40 ± 0.01	0.25 ± 0.01	0.50 ± 0.01	0.24 ± 0.01
	500		0.19 ± 0.03	0.17 ± 0.03	0.11 ± 0.02	0.21 ± 0.03	0.11 ± 0.02
			0.40 ± 0.01	0.35 ± 0.01	0.23 ± 0.01	0.48 ± 0.01	0.23 ± 0.01
6DWI	0		0.18 ± 0.05	0.18 ± 0.01	0.12 ± 0.01	0.22 ± 0.01	0.11 ± 0.01
			0.39 ± 0.01	0.37 ± 0.01	0.26 ± 0.01	0.49 ± 0.01	0.24 ± 0.01
	200		0.16 ± 0.01	0.17 ± 0.01	0.11 ± 0.01	0.20 ± 0.02	0.10 ± 0.01
			0.35 ± 0.01	0.36 ± 0.01	0.22 ± 0.01	0.45 ± 0.01	0.21 ± 0.01
	500		0.15 ± 0.01	0.15 ± 0.03	0.10 ± 0.01	0.19 ± 0.01	0.9 ± 0.02
			0.33 ± 0.01	0.33 ± 0.01	0.21 ± 0.01	0.43 ± 0.01	0.18 ± 0.01

2DWI	0	Mahogany	0.17 ± 0.01	0.16 ± 0.3	0.12 ± 0.01	0.21 ± 0.01	0.11 ± 0.02
			0.33 ± 0.01	0.36 ± 0.01	0.25 ± 0.01	0.44 ± 0.01	0.24 ± 0.01
	200		0.16 ± 0.01	0.15 ± 0.02	0.11 ± 0.00	0.20 ± 0.03	0.10 ± 0.02
			0.31 ± 0.01	0.34 ± 0.01	0.26 ± 0.01	0.41 ± 0.01	0.21 ± 0.01
	500		0.15 ± 0.01	8.14 ± 0.03	0.10 ± 0.03	0.19 ± 0.02	0.09 ± 0.01
			0.30 ± 0.01	0.29 ± 0.01	0.20 ± 0.01	0.39 ± 0.01	0.18 ± 0.01
6DWI	0		0.16 ± 0.01	0.15 ± 0.02	0.11 ± 0.01	0.20 ± 0.04	0.10 ± 0.01
			0.32 ± 0.01	0.32 ± 0.01	0.25 ± 0.01	0.40 ± 0.01	0.20 ± 0.01
	200		0.14 ± 0.03	0.13 ± 0.01	0.10 ± 0.02	0.19 ± 0.02	0.9 ± 0.03
			0.30 ± 0.01	0.27 ± 0.01	0.20 ± 0.01	0.38 ± 0.02	0.18 ± 0.01
	500		0.12 ± 0.01	0.12 ± 0.03	0.9 ± 0.03	0.17 ± 0.01	0.8 ± 0.02
			0.25 ± 0.01	0.25 ± 0.01	0.19 ± 0.01	0.35 ± 0.01	0.16 ± 0.01

	*FLZ*		0.15 ± 0.01	0.20 ± 0.01	0.13 ± 0.01	0.21 ± 0.01	0.10 ± 0.01
			0.28 ± 0.03	0.33 ± 0.01	0.22 ± 0.03	0.33 ± 0.01	0.21 ± 0.01
	*KTZ*		0.10 ± 0.01	0.21 ± 0.01	0.21 ± 0.01	0.19 ± 0.01	0.20 ± 0.01
			0.20 ± 0.01	0.40 ± 0.01	0.40 ± 0.01	0.42 ± 0.01	0.40 ± 0.01

## Data Availability

All data used to support the findings of this study are included within the article.
